# Intracultural Differences in Local Botanical Knowledge and Knowledge Loss among the Mexican Isthmus Zapotecs

**DOI:** 10.1371/journal.pone.0151693

**Published:** 2016-03-17

**Authors:** Alfredo Saynes-Vásquez, Heike Vibrans, Francisco Vergara-Silva, Javier Caballero

**Affiliations:** 1 Postgrado en Botánica, Campus Montecillo, Colegio de Postgraduados, Montecillo, Texcoco, México; 2 Jardín Botánico, Instituto de Biología, Universidad Nacional Autónoma de México, Ciudad Universitaria, Distrito Federal, Mexico; College of Agricultural Sciences, UNITED STATES

## Abstract

This study reports on the socio-demographic and locality factors that influence ethnobiological knowledge in three communities of Zapotec indigenous people of the Isthmus of Tehuantepec, Mexico. It uses local botanical nomenclature as a proxy for general ethnobiological knowledge. In each of these communities (one urban and two rural), 100 adult men were interviewed aided with a field herbarium. Fifty had a background in farming, and 50 worked in the secondary or tertiary sector as their main economic activity, totaling 300 interviews. Using a field herbarium with samples of 30 common and rare wild regional species, we documented visual recognition, knowledge of the local life form, generic and specific names and uses (five knowledge levels measuring knowledge depth). The relationship between sociodemographic variables and knowledge was analyzed with simple correlations. Differences between the three communities and the five knowledge levels were then evaluated with a discriminant analysis. A general linear analysis identified factors and covariables that influenced the observed differences. Differences between the groups with different economic activities were estimated with a t-test for independent samples. Most of the relationships found between sociodemographic variables and plant knowledge were expected: age and rurality were positively related with knowledge and years of formal schooling was negatively related. However, the somewhat less rural site had more traditional knowledge due to local circumstances. The general linear model explained 70–77% of the variation, a high value. It showed that economic activity was by far the most important factor influencing knowledge, by a factor of five. The interaction of locality and economic activity followed. The discriminant analysis assigned interviewees correctly to their localities in 94% of the cases, strengthening the evidence for intracultural variation. Both sociodemographic and historic intracultural differences heavily influence local knowledge.

## Introduction

Ethnobotanical and other ethnobiological studies of intracultural differences have focused on sociodemographic factors. For example, traditional knowledge varies with age [1–4], gender [5–7], family relationships [8,9] and the nature of the cultural domain. Anthropologists have long emphasized intracultural variability, diversity or heterogeneity due to historical processes and geographical circumstance [6,10,11]. Ethnobiologists have rarely studied this factor.

Ethnobiologists and sociocultural anthropologists characterize culture in various ways. These characterizations generally emphasize the intentional or unintentional transmission of attitudes, behaviors and knowledge, a process that involves experience and observation as well as imitation and instruction. Defining culture is commonly recognized as a difficult task in anthropological theory (e.g. [12]). Here, we use a cognitive approach and define culture as whatever one has to know or believe in a community in order to operate in a manner acceptable to its members; it includes knowledge, belief, art, law, morals, custom and any other capabilities and habits acquired through imitation and learning, not heredity. This is based on the definitions of Goodenough [13] and Tylor [14]

Within a given cultural unit or subunit, there are idiosyncratic differences between individuals. Edward Sapir noted in 1930 that not all people, even in small-scale societies, act or behave in the same way; even earlier, in 1884, Dorsey described the same phenomenon in his study of the Omaha (cited in [10]). A debate exists on the limits of these subgroups: at what point is knowledge acquisition and/or construction an idiosyncratic individual matter, or the result of a group process? Some investigators minimize the influence of these differences, but others consider them so important as to make the culture concept unhelpful, if it obscures the importance of the individual dimension [11]. However, Weller *et al*. [15] showed that despite strong sociodemographic differences, some basic consensus can usually be identified within cultural collectives. For example, belief in culturally defined illnesses, such as *empacho* in Latin America, is often widely shared among large groups of individuals.

If a culture is under strong outside influence from another culture, additional factors determine the dynamics of knowledge acquisition/construction and loss. For instance, religious affiliations may change, resulting in modified values, perception, and relationships [16]. Dominant cultures impose changes in taste and values, linguistic competence, and traditional agricultural practices. These changes lead to knowledge loss, particularly in those domains associated with vegetation and ecology. These knowledge-related processes are modulated by local language competence (e.g. people who do not speak the dominant language take longer to change), economic activity, salience or importance of the knowledge, and formal education [17–23]. Cultures can vary considerably in their response to outside pressure [24,25].

Both static and dynamic knowledge effects are often evident through classifications and nomenclature for concepts and objects. These differences can be measured and analyzed. This type of data helps to understand and quantify the dimension and role of cultural identity, differences and transmission, and the direction of cultural change [26–28].

Knowledge held by people of their surroundings (plants, animals, ecosystems, etc.) can be accumulated throughout their histories [29,30]. This local knowledge crucially depends on continuous interaction, particularly of children and young adults, with their surroundings and their elders [31–33]. Globalization, insensitive educational systems, land right modifications, changes in economic activities and geographic and linguistic displacement modify these interactions and relationships, and interrupt transmission [20,34–39]. All of these factors, alone or in diverse combinations, in principle, exert pressure on this knowledge, often making it appear less valuable. In his classical ethnobiological work, Berlin [40] discussed the loss of ethnobiological vocabulary, as certain activities lose cultural importance. However, these influences are frequently studied separately, and their relative contribution is still widely unknown.

Archeologists (e.g. Marcus and Flannery, [41]) and sociocultural anthropologists (e.g. Peterson Royce [42]; Whitecotton [43]; Saynes-Vázquez [44]) have studied the Zapotec cultural area–located in the state of Oaxaca, Mexico–for decades. An early ethnobotanical reference is the work on botanical symbolism by Reko [45]. Ethnobiological work has shown that Zapotecs have a profound knowledge of their natural surroundings and acquire their knowledge early [46–49]. In recent years, knowledge loss induced by cultural change has accelerated; see, for instance, the study of Cortés-González [6] for Nizanda, whose people speak the same Zapotec variant as people in our study area. Frei and coauthors [50–53] have studied medicinal plants of the Zapotecs of the Sierra Madre del Sur extensively. However, the focus of these works was pharmacological properties, and not local nomenclature and classification.

The Zapotec plant and animal classification systems have been explored repeatedly [46,54–58]. All six levels of folk classifications proposed by Berlin [59]–namely kingdom, life form, intermediate, generic, specific and varietal–have been found in Zapotec groups. Depending on their region of origin, Zapotecs distinguish five to thirteen life forms [46,55–58,60]. Most plant names are simple generic names (consisting of one word), but some have modifiers, that is, are composite or secondary generics; these are called specific names in this study.

This work studies the response of the Isthmus of Tehuantepec Zapotecs to a complex and relatively well-documented historical process involving both imposition and dominance, as well as resistance and syncretism (e.g. [61–65]). Zapotec botanical nomenclature serves as a metric and proxy for general ecological knowledge. To exclude the well-known influence of gender, we worked only with men.

We attempt to quantify the relative importance of various sociodemographic factors known to influence traditional ecological knowledge, such as economic activity, schooling/formal education, age, language competence, locality and their interactions. We expect farmers and more rural communities to conserve significantly more traditional plant knowledge than people in other sectors, and the urbanized population. Additionally, we expect to identify other factors that influence knowledge within these groups, particularly those related to locality and local history. The combination of these data—sociodemographic and locality-focused—helps us to characterize the drivers of ecological knowledge and intracultural differences.

## Materials and Methods

### The study area

**Fig 1:**
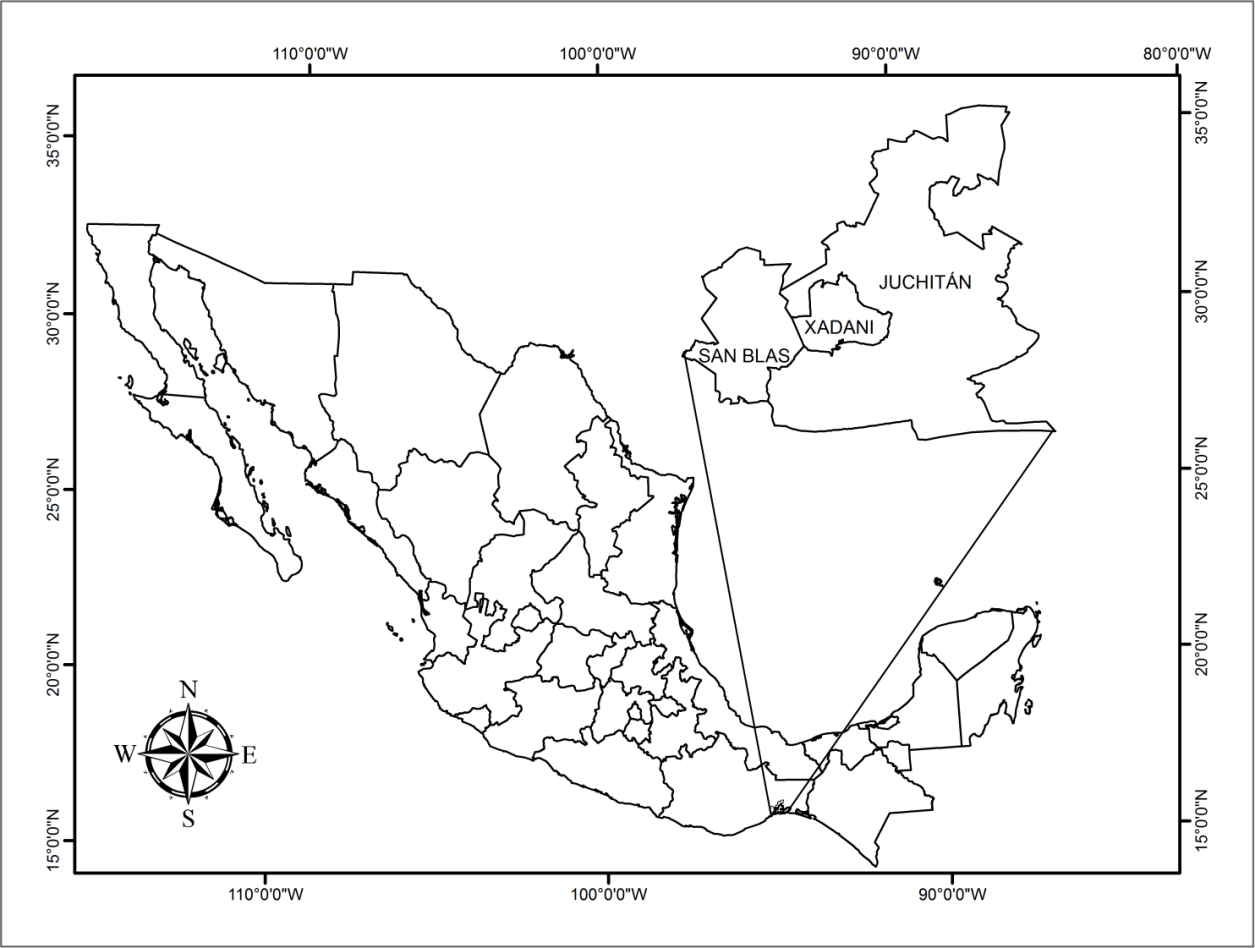
Location of the study sites. Map reproduced from Saynes-Vásquez et al. [26] under a Creative Commons Licence 2.0.

The study region comprised the municipalities of Juchitán de Zaragoza (16° 25´ 58.4” N; 95° 01´ 19.1” W), San Blas Atempa (16° 19´ 37” N; 95° 13´ 39” W) and Santa María Xadani (16° 21´ 36” N; 95° 01´ 11” W), all located in the Isthmus of Tehuantepec in southern Oaxaca, Mexico (Fig 1).

The sites broadly share both vegetation types (tropical dry forests) and the Isthmus Zapotec language, part of the Otomangue language family, with some dialect variations [66]. Zapotec is still widely spoken in daily life in the region with at least 70% of the population competent, even in an urban context. These municipalities represent a sociocultural gradient with respect to urbanization and formal education, as used by Thompson [17], Norton *et al*. [18], Diamond and Bishop [19], Turner *et al*. [20], Zent [27,28], Kakudidi [21], Maffi [22] and Martínez-Ballesté *et al*. [23]. Table 1 shows the main indicators of the sociocultural gradient, such as the proportion of the population who are farmers, and years of formal education.

**Table 1:** Levels of linguistic competence and formal education in the study area reflect the gradient of cultural displacement.

Municipality	Proportion (%) of Zapotec speakers	Proportion (%) of monolingual Zapotec speakers	Proportion (%) of the population that can read and write Spanish (age over 15)	Average years of formal education	Proportion (%) of the population whose main income derives from primary activities
Juchitán de Zaragoza	70	8.8	52.1	6.3	13.9
San Blas Atempa	92.8	24.9	40	3.6	33.8
Santa María Xadani	97.7	34	32.1	3.4	35

The proportion of people that speak an indigenous language is relatively high in comparison with most present-day Mexican municipalities. The levels of monolingüism have descended in the last decades, though perhaps not as much as in other regions: in 1950, about one-quarter of the townspeople of Juchitán were monolingual (27%), while the more rural municipalities had much higher proportions: 72% in San Blas Atempa and 83% in Santa María Xadani[67–69]. Today, somewhat less than one-half of these proportions continue to speak only Zapotec. The region is well-communicated by the Pan-American Highway and various federal and state highways. All of the communities have schools, hospitals, telephone and cell phone service, and partial internet access.

Juchitán is an important regional market town, with some manufacturing, but farmers also live there. In the two small towns of our study area, agriculture remained an important activity, but there were also many people employed in the secondary and tertiary sector. Land ownership was originally communal, as in many indigenous communities in Mexico, but large parts have been privatized illegally in the last decades [70–72]. Land ownership has been a source of continuous conflict, sometimes violent, stoked even more by the arrival of the eolic energy industry in the region.

San Blas is a small town relatively close to a large refinery built in 1979; many inhabitants work there. Workers need fluency in Spanish, as most of the administration personnel is from elsewhere. However, the community still has a functional communal land administration, with regular assemblies and a Comisariado de Bienes Comunales (the Commissary of Communal Property), in contrast to the other two sites.

Santa María Xadani is also quite close to a city (Juchitán) and many people travel there daily for work or study. However, the work available there is in the commercial and low-level trades sector, where Zapotec is the *lingua franca* [44]. These factors explain the differences in the cultural indicators (language competence, etc.) contained in Table 1, and make Xadani slightly more traditional and rural.

Both Santa María Xadani and San Blas Atempa have a peculiarity: a small hill with relatively conserved vegetation within the town, which has ceremonial significance, but also functions as a common for foraging goats, etc. Most people walk in them several times a year, and they thus serve as a point of contact with natural vegetation even for those people with no interest in agriculture. Also, all three communities organize yearly pilgrimages to sacred sites located between 3 and 11 km from the community centers, and participants always cross natural vegetation.

### Species selection and field catalog

For the interviews, we selected 30 species (Table 2), all of which occurred in a large tropical dry forest named Igú of about 24 km^2^ that was located in the confluence of the three studied communities, and accessible for the population of all of them. The floristics, ethnobotany and local plant nomenclature of this forest had been studied from 2005 onwards, though the results were never published. Each life form recognized by the studied population was represented by six species. The life forms were trees (*yaga*), shrubs (considered “little trees” in the local perception, *yaga huini*), vines (*luba´*), herbs (*guishi*) and a local category called guie', which means flower. Species in this group often have colorful flowers and the local Zapotecs distinguish them from other life forms.

**Table 2:** Species selected for the interviews on botanical knowledge. Parts of this table have been published previously in Saynes-Vásquez et al. [26] under a Creative Commons Licence 2.0; the uses are added as they are relevant for this paper.

Family	Species	Zapotec name	Uses	
Amaranthaceae	*Amaranthus spinosus* L.	Balaadxi gui'chi' (H)	Animal fodder		
Apocynaceae	*Marsdenia coulteri* Hemsl.	Luba' biñaa (V)	Food		
Apocynaceae	*Plumeria rubra* L.	Guie' chachi (G)	Ritual		
Bignoniaceae	*Crescentia alata* Kunth	Bitu xhiga gui´xhi (S)	Handicrafts		
Bignoniaceae	*Mansoa hymenaea* (DC.) A.H. Gentry	Luba' bete (V)	Medicinal		
Burseraceae	*Bursera schlechtendalii* Engl.	Yalaguitu (T)	Medicinal		
Combretaceae	*Combretum fruticosum* (Loefl.) Stuntz	Luba' begu (V)	Ornamental		
Cleomaceae	*Polanisia viscosa* (L.) DC.	Stope gui'xhi' (H)	Medicinal		
Commelinaceae	*Commelina erecta* L.	Guie'duza (G)	Medicinal		
Cucurbitaceae	*Ibervillea* sp. nov.	Luba' cuba, Luba' manzanina, Luba' melón gui'xhi' (V)	Animal fodder		
Euphorbiaceae	*Croton niveus* Jacq.	Copachil (S)	Construction		
Euphorbiaceae	Chamaesyce cf. Dioeca (Kunth) Millsp.	Pichinchi yuu (H)	Medicinal		
Celastraceae	*Hippocratea excelsa* Kunth	Luba' biichi (V)	Ritual		
Anacardiaceae	*Amphipterygium adstringens* (Schltdl.) Standl.	Yaga yala (T)	Medicinal		
Fabaceae	*Aeschynomene americana* L.	Yaga tama (S)	Fodder		
Fabaceae	*Apoplanesia paniculata* C. Presl	Guie' bi'chi' (G)	Ornamental		
Fabaceae	*Diphysa minutifoli*a Rose	Guiiña' bidxi (S)	Firewood		
Fabaceae	*Lonchocarpus emarginatus* Pittier	Guie' gade (G)	Firewood		
Fabaceae	*Microlobius foetidus* (Jacq.) M. Sousa & G. Andrade	Biquiiche dxa (T)	Firewood		
Fabaceae	*Mimosa acantholoba* (Humb. & Bonpl. ex Willd.) Poir.	Chumaga o Guichi xhi gueza (S)	Firewood,		
Fabaceae	*Senna atomaria* (L.) H.S. Irwin & Barneby	Besa duni (T)	Firewood		
Fabaceae	*Senna skinneri* (Benth.) H.S. Irwin & Barneby	Guie' bizu, Bara seda (G)	Firewood		
Fabaceae	*Prosopis juliflora* (Sw.) DC.	Yaga bii (T)	Firewood, construction		
Malpighiaceae	*Malpighia emarginata* DC.	Combriu (S)	Food		
Malvaceae	*Gossypium* aff. *aridum* (Rose & Standl.) Skovst.	Xiaa gui'xhi'(H)	Medicinal		
Portulacaceae	*Portulaca oleracea* L.	Xedxe (H)	Animal fodder		
Rhamnaceae	*Ziziphus amole* (Sessé & Moc.) M.C. Johnst.	Xuba beza (T)	Food		
Sapindaceae	*Serjania goniocarpa* Radlk.	Luba' golondrina (V)	Medicinal		
Scrophulariaceae	*Capraria biflora* L.	Bitiaa gui' xhi' (H)	Domestic tool		
Primulaceae	*Jacquinia pungens* A. Gray	Guie' zee (G)	Ornamental		

(T) = tree, (S) = shrub, (V) = vine, (H) = herb, (G) = guie'

**Fig 2:**
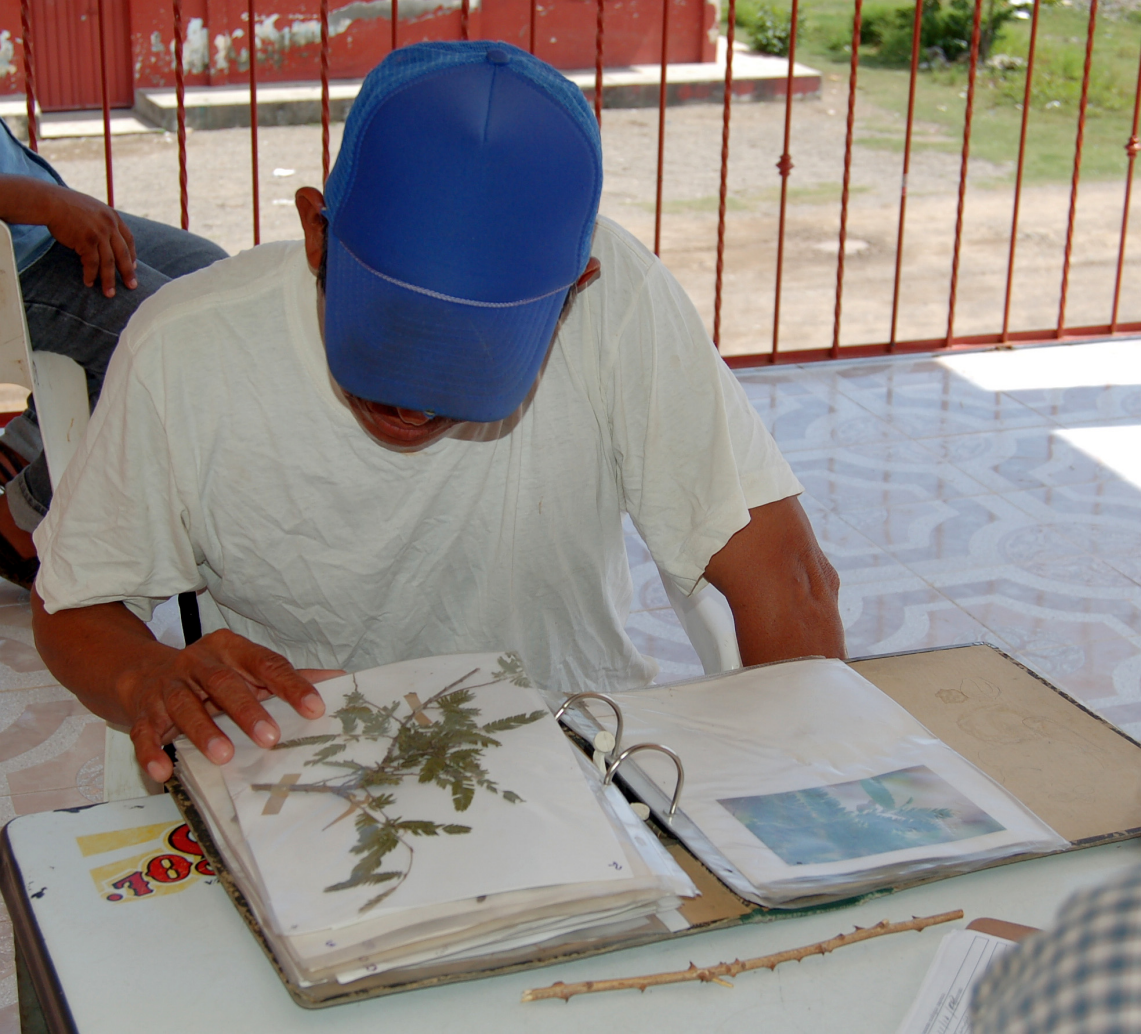
Interview with the field herbarium.

For selecting the woody plants (trees, shrubs, vines), we used survey data (unpublished) of thirty 50 x 2 m transects (with the method proposed by Trejo and Dirzo [73]). For each life form, we selected two species with a high ecological importance value, two with a medium value and two with a low value; another criterion was that they have a Zapotec name. The herbs were selected at random, but with the condition that they have a species-specific Zapotec name. We made a field herbarium of the thirty species, with a dried specimen and quality photographs of the habit, stem, flowers and fruit [74,75] (Fig 2). The names were transcribed according to Pickett [76].

### The interviews

We interviewed only adult men (average age 48.6, minimum 19, maximum 78), in order to exclude one common source of knowledge variability, gender. Also, we avoided first-degree relatives. Another condition was that their parents spoke Zapotec. In each community, we interviewed 100 persons, for a total of 300. At each site, half of the interviewees worked in the primary sector (farmers, hunters or firewood collectors), and the other half were urbanized workers, traders or professionals; that is, they worked in the secondary or tertiary sector. The field work was conducted between 2006 and 2009.

It was not possible to obtain a random sample of the population, for several reasons: large communities, intracommunity political conflict and lack of a reliable list of private and communal landowners. To obtain collaboration of the various political fractions, we attended five to seven communal and political meetings of the different groups in each community. We explained the project and requested collaboration for the interviews. The volunteers were interviewed either in their home or their place of work (fields, offices, stores, etc.), always alone, and in Zapotec, if they spoke the language. Informed consent was obtained again orally and individually before each interview, and anonymity was assured. No names or identifying information was recorded (see S1 Fig for the form). Representatives of the local communities extended a formal letter that a specific permit for this work was not required (S2 Fig). The plants used for the interviews had been collected for a previous project under an institutional collection permit to the National Herbarium, MEXU.

The interviews were semi-structured and based on the field herbarium mentioned above (Table 2, Fig 2 and S2 Fig). For each plant, respondents were asked the following questions: Have you seen the plant? This was considered the most general and superficial level of knowledge. Is it a tree, shrub, liana, herb or guie'? What is its name? Is it used for something? These questions were aimed to reflect depth or sophistication of knowledge, in this order. We also asked for descriptions of the plant and its habitat in order to confirm the identification. Based on these questions, we distinguished the following knowledge levels (reflecting knowledge depth) for each plant: 1. Visual recognition; 2. Plant form; 3. Generic name; 4. Specific name and 5. Use.

Socioeconomic data obtained included age, main economic activity, years of formal schooling and competence in the Zapotec language. Language competence was classified on a 1–5 scale (modified from Zent [27]): 1 = Zapotec monolingual, 2 = speaks Zapotec, understands Spanish; 3 = bilingual; 4 = understands Zapotec and speaks Spanish. There were no interviewees in the possible category 5, monolingual in Spanish, even though they were not excluded intentionally.

### Data analysis

The Global Index of each interviewee was the sum of all questions answered correctly. That is, each of the five questions mentioned above could result in 1 point; the maximum level was 150 points for 30 species x 5 answers. If someone could not recognize any plant, he would have a score of 0. Although we present the original data for some descriptive statistics, we normalized the data for several statistical analyzes according to Freeman and Tukey (in [77]).

Discriminant analysis elucidated the differences between municipalities and activities. Also, the relationship between sociodemographic variables and knowledge categories was analyzed by a general linear analysis, which combined an ANOVA with correlation analysis. We used SPSS v. 21.0 and Statistica v. 7 for statistical calculations.

## Results

This study investigated the relationship of plant knowledge and depth with the following factors: main economic activity, age, language competence, years of formal schooling, locality/rurality. First, we discuss each factor separately, and then we examine their interactions and their relative importance.

### Plant knowledge and the main economic activity

Not surprisingly, people whose main occupation was related to primary activities are much more knowledgeable about plants than those with secondary or tertiary activities. Table 3 shows the descriptive statistics for the knowledge exhibited by type of economic activity and locality of the interviewee. In both Juchitán and San Blas, farmers had about twice as much knowledge as people with other activities, as measured by the Global Index. The absolute scores of the farmers were rather similar to each other (though statistically different by location, as we will show further down). However, in the most rural community, Santa María Xadani, farmers had about the same knowledge level as in the other sites, but non-farmers knew about three-quarters as much. The same pattern surfaces with more sophisticated knowledge, such as plant names or uses. The differences in knowledge between farmers and non-farmers are highly significant with a *t*-test (p<0.0001) (see S1 Table).

**Table 3:** Descriptive statistics for the plant knowledge competence exhibited by type of main economic activity and locality of the interviewee.

Locality/Sector	Competence	Minimum	Máximum	Mean	Std. Dev.	Coeff.Var. (%)
Juchitán de Zaragoza	Visual recognition	21	30	27.64	1.93	6.96
Primary sector	Plant form	21	30	27.56	1.93	7
	Generic name	19	30	26.04	2.47	9.47
	Specific name	15	30	24.18	3.64	15.03
	Use	15	28	22.78	2.96	13.01
	Global Index	93	148	128.2	11.45	8.93
Juchitán de Zaragoza	Visual recognition	4	28	16.5	6.41	38.82
Secondary and Tertiary sector	Plant form	2	26	15.4	6.85	44.51
	Generic name	0	25	11.18	7.05	63.09
	Specific name	0	23	8.24	6.72	81.6
	Use	1	22	9.82	6.1	62.12
	Global Index	9	122	61.14	31.77	51.97
San Blas Atempa Primary sector	Visual recognition	25	30	28.9	1.47	5.1
	Plant form	25	30	28.86	1.51	5.24
	Generic name	18	30	27.22	2.76	10.13
	Specific name	16	30	26.62	3.1	11.66
	Use	17	30	24.48	2.84	11.59
	Global Index	105	150	136.08	10.89	8
San Blas Atempa Secondary and Tertiary sector	Visual recognition	7	27	18.62	4.36	23.39
	Plant form	7	27	18.16	4.27	23.5
	Generic name	2	21	13.32	4.55	34.17
	Specific name	0	21	10.54	4.89	46.37
	Use	2	21	11.44	3.96	34.57
	Global Index	29	110	72.08	20.36	28.25
Santa María Xadani Primary sector	Visual recognition	25	30	28.12	1.47	5.21
	Plant form	24	30	27.96	1.63	5.82
	Generic name	19	29	26.18	2.28	8.72
	Specific name	16	29	25.08	3.13	12.48
	Use	17	29	24.14	2.17	8.98
	Global Index	111	147	131.48	8.96	6.81
Santa María Xadani Secondary and Tertiary sector	Visual recognition	13	29	22.56	3.45	15.28
	Plant form	13	29	22.4	3.41	15.2
	Generic name	11	28	19.62	4.19	21.38
	Specific name	6	26	16.48	5.32	32.3
	Use	6	25	16.68	4.37	26.19
	Global Index	51	134	97.74	19.47	19.92

Variation of knowledge was lower in the group with primary activities, compared with the group with activities unrelated to natural resources, according to the Global Index. This was true in all locations, and variation was highest in the inhabitants of the city of Juchitán (52%). In the smaller towns, variation was about 20 and 30%. Variation also increased with knowledge depth or sophistication in all groups.

### Plant knowledge and age

**Fig 3:**
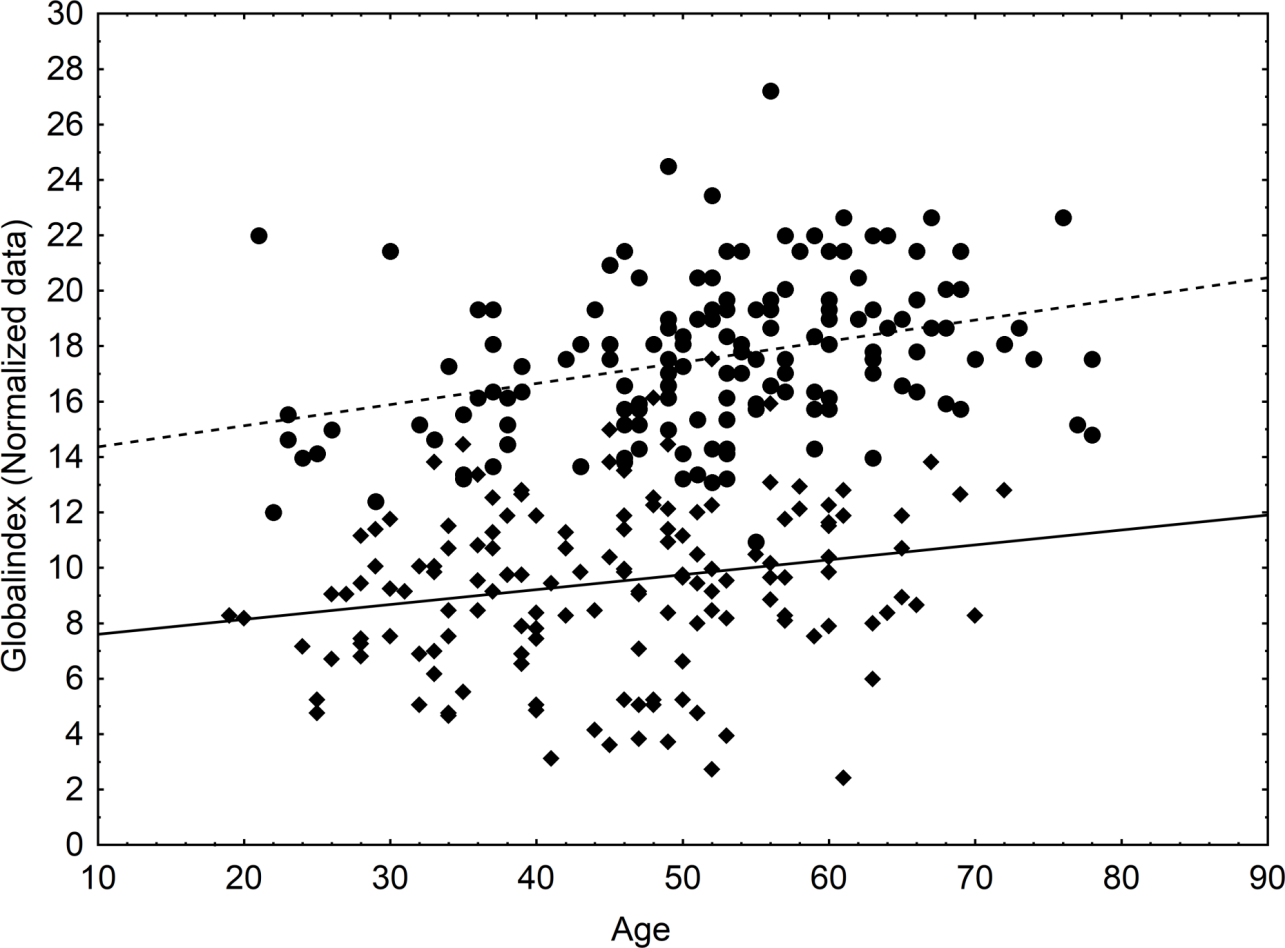
Relationship of age, knowledge and economic activity. Dotted line: Activity 1, r^2^ = 0.1105, p = <0.001; solid line: Activity 2 and 3, r^2^ = 0.0420, p = 0.012.

Knowledge increased with age, significantly, but only slightly. However, the relationship was stronger for farmers (Primary sector in Fig 3), and much more variable for people with other activities (Secondary and tertiary sector).

### Plant knowledge and language competence

The analysis of the relationship between language competency and plant knowledge showed the expected positive correlation (Table 4). However, two-thirds of our interviewees were in the bilingual category, and there were relatively few people in some groups, so the data were only moderately informative. The differences between groups were statistically highly significant with an ANOVA (p = < 0.001; see S2 Table).

**Table 4:** The descriptive statistics for the knowledge exhibited by type of Zapotec language competence of the interviewee.

Language competence	Knowledge	N	Minimum	Maximum	Mean	Std. Deviation	Variance
1	Visual recognition	38	21	30	28.18	1.915	3.668
	Plant form	38	21	30	28.08	1.95	3.804
	Generic name	38	19	30	26.74	2.298	5.28
	Specific name	38	15	30	25.58	3.193	10.196
	Use	38	17	29	23.87	2.495	6.225
	Global Index	38	93	147	132.45	10.901	118.849
2	Visual recognition	51	16	30	26.84	3.844	14.775
	Plant form	51	16	30	26.84	3.844	14.775
	Generic name	51	11	30	24.94	4.781	22.856
	Specific name	51	8	30	23.84	5.64	31.815
	Use	51	9	30	22.31	4.823	23.26
	Global Index	51	66	150	124.78	22.28	496.413
3	Visual recognition	206	4	30	22.44	6.121	37.467
	Plant form	206	2	30	22.02	6.408	41.058
	Generic name	206	0	30	18.84	7.477	55.911
	Specific name	206	0	30	16.34	8.311	69.066
	Use	206	1	29	16.51	7.054	49.763
	Global Index	206	9	149	96.16	34.402	1183.482
4	Visual recognition	5	9	13	11	1.581	2.5
	Plant form	5	7	11	8.8	2.049	4.2
	Generic name	5	0	3	1.8	1.095	1.2
	Specific name	5	0	1	0.4	0.548	0.3
	Use	5	3	5	4	1	1
	Global Index	5	2	30	26	4.301	18.5

1 = monolingual in Zapotec; 2 = understands but does not speak Spanish; 3 = bilingual; 4 = understands but does not speak Zapotec.

### Plant knowledge and formal schooling

**Fig 4:**
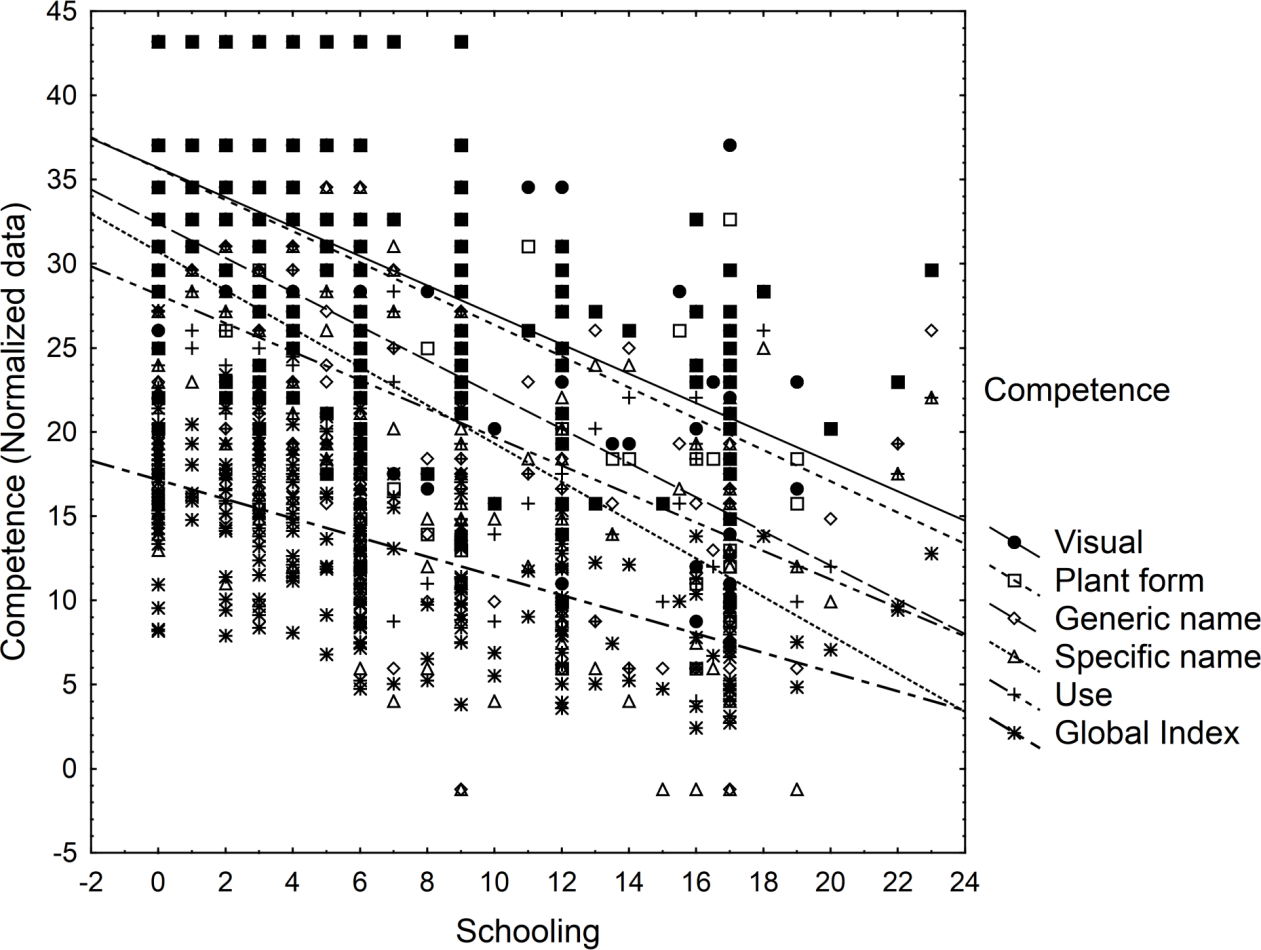
Relationship between years of schooling and different levels of competence or knowledge. Visual recognition: r^2^ = 0.352, p = <0.001; Plant form: r^2^ = 0.3742, p = <0.001; Generic name: r^2^ = 0.4077, p = <0.001; Specific name: r^2^ = 0.426, p = <0.001; Use: r^2^ = 0.43, p = <0.001; Global Index: r^2^ = 0.42, p = <0.001.

People with more formal education know less about plants. This negative relationship is highly significant and can be shown by both total knowledge (Global Index; r^2^ = 0.42, p = <0.001; Fig 4) and depth of knowledge. The correlation coefficient is higher for more sophisticated knowledge. Schooling explains 35.2% of the variation for simple visual recognition, 42.6% for the specific name and 43% for use (Fig 4).

### Plant knowledge and location

**Fig 5:**
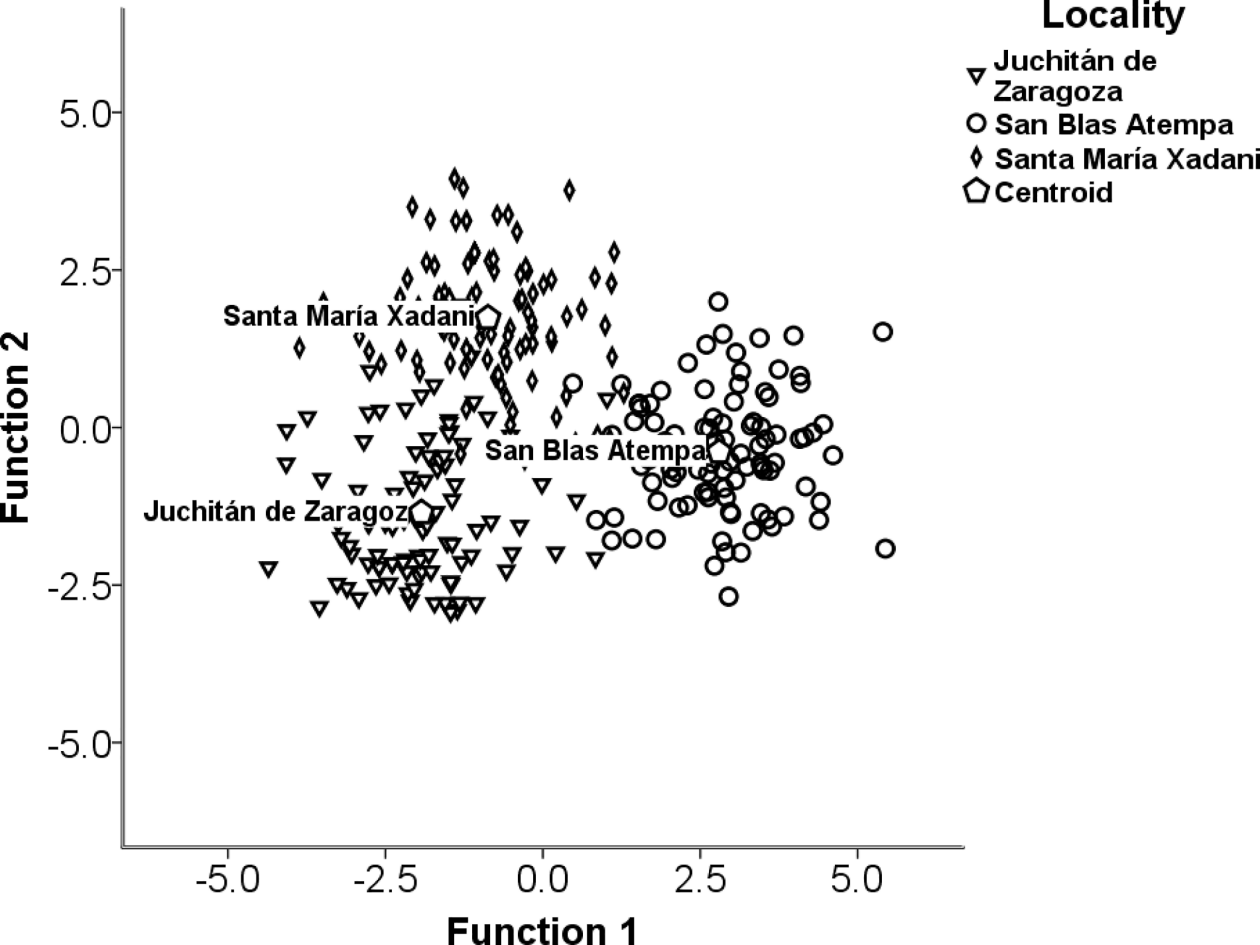
Discriminant analysis of the relationship between locality and knowledge. Inverted triangle: Juchitán de Zaragoza; diamond: Santa María Xadani; circle: San Blas Atempa; pentagon: entroid.

Knowledge depends not only on one's economic activity but also on one's locality. Using the five knowledge levels, discriminant analysis correctly assigned the interviewees to their groups in 94.3% of the cases (Wilks' Lambda = 0.072; χ^2^ = 600.175 df = 276; p = <0.001; Fig 5).

**Fig 6:**
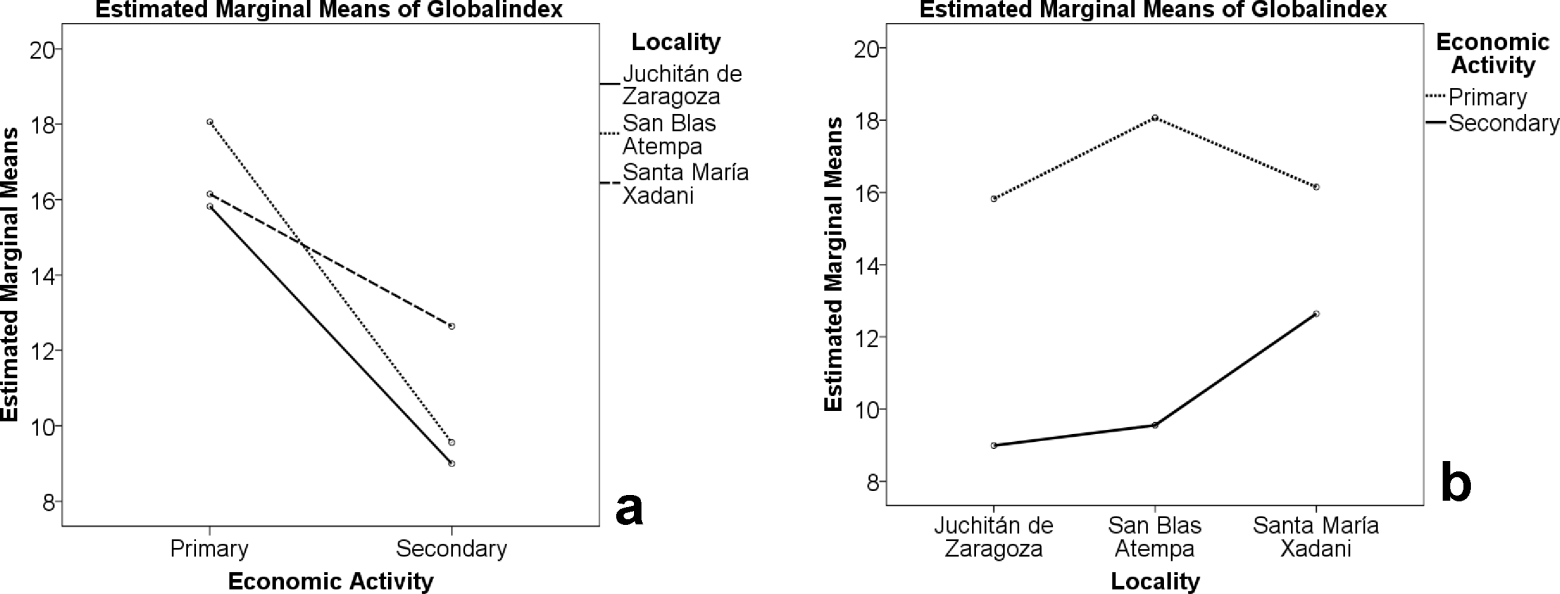
The relationship between knowledge and locality, considering different types of economic activity. a: Economic activity and knowledge, showing the three localities and their different slopes, indicating different rates of knowledge loss. b: Locality and knowledge, showing the different behavior by economic activity.

As expected, the town population of Juchitán knew less than rural interviewees. This applied to people with primary as well as to those with secondary activities. The study site with the highest average knowledge by farmers was San Blas Atempa, that is, the somewhat less rural site, according to the demographic indicators. Fig 6 shows the marginal mean of the Global Index for the three study sites and the two types of economic activity; the slope of the line indicates the rate of knowledge loss. It shows that knowledge loss is much slower in the most rural area, Santa María Xadani, but faster at the intermediate site, San Blas, than in the other two.

### Relative importance of the sociodemographic factors

All studied variables were significant (type of economic activity, years of schooling, interaction between locality and economic activity, age, locality and fluency in Zapotec). With the Global Index as the dependent variable, a general linear analysis resulted in a model with high explicative power (corrected R^2^ = 77.6%; Table 5). The different levels of knowledge showed similar results, with the levels of explanation varying between 69.5 and 75.6%. (see S3 Table). However, sociodemographic factors influenced knowledge and depth of knowledge to different degrees. The Partial Squared Eta shown in Table 5 for the Global Index quantifies the relative importance of these factors.

**Table 5:** The relationship between the Global Index knowledge and the sociodemographic variables. Analyzed with a general linear analysis of between-subject effects and its statistical significance. It shows the relative contribution of each variable (Partial Eta Squared) (for the other levels of knowledge, see S3 Table).

Source	Type III Sum of Squares	Df	Mean Square	F	Sig.	Partial Eta Squared
Corrected Model	5858.936^a^	8	732.367	130.825	.000	.782
Intercept	300.849	1	300.849	53.742	.000	.156
Age	116.674	1	116.674	20.842	.000	.067
Schooling	181.466	1	181.466	32.416	.000	.100
Speak Zapotec	30.212	1	30.212	5.397	.021	.018
Locality	200.737	2	100.368	17.929	.000	.110
Economic activity	1691.070	1	1691.070	302.081	.000	.509
Locality * Economic activity	319.687	2	159.844	28.553	.000	.164
Error	1629.038	291	5.598			
Total	62463.746	300				
Corrected Total	7487.974	299				

^a^ r^2^ = 0.782 (Adjusted r^2^ = 0.776).

The analysis of the individual factors showed the expected general results. However, the relative importance of the factors was notable. The influence of the economic activity on total knowledge (Global Index) was five times greater than locality or formal schooling, 7.6 times as important as age and 28 times as important as Zapotec language competency. However, the interaction of economic activity with locality was in second place of importance in most levels of knowledge. The data for the other levels of knowledge can be found in the S3 Table.

## Discussion

### Plant knowledge and simple sociodemographic factors

Mostly, the results support previous work. They showed positive correlations between plant knowledge and age [1–4,27,28,78], indigenous language knowledge [28,36,79] and rurality [78,80], as well as a negative correlation with years of formal schooling [6,28]. Occupation has also been cited previously as an important factor [17,23]. Saynes-Vásquez et al. [26] have proposed an index of cultural displacement that integrates all these variables, finding a significant negative correlation between the index and knowledge at all levels. Farmers are more exposed to natural habitats, and partially obtain their livelihoods from them, which means they are interested [17,23].

Shenton el al. [33] found that rural populations of Chiapas Maya knew more plants than the more urbanized people, but the authors were not able to say if this was due to exposure or to changed cultural patterns. Another study [38,39], found cultural influences to be decisive: of three groups—longterm indigenous residents, immigrated ladinos with close contact to the longterm residents, and recently immigrated indigenous people, the ladinos knew more than the constantly exposed, but recently immigrated population. Ross et al. [31,32] also interpreted their results of the study of the plant knowledge of Lacandon youths as representing a cultural change, rather than lack of exposure. In our study, with a more homogenous and longterm resident population, we found that the importance of economic activity–that is, exposure—was overwhelming, even for people living in rural environments. Cultural factors (locality) were also significant, but not nearly as important as occupation.

The relatively low inclination of the relationship between age and knowledge, though significant, can be explained by the fact that we interviewed only adults. Most studies have shown that people acquire knowledge of natural domains at a young age [11, 28,37].

Formal schooling influences plant knowledge in different ways. First, it reduces the time children are exposed to plants; they have less time to accompany their parents to work and to help them collect useful plants. Second, the dominance of the national language, Spanish, in school reduces their competence in and exposure to their native language, which is the primary vehicle for traditional knowledge transmission. Data that show that in mestizo Spanish-language communities, formal schooling has no influence on the knowledge of uses of plants [80], support this reasoning.

The age at which children enter school is also relevant. If they start school with primary education at six years of age, they will already have substantial competence in their native language. However, if they start with preschool at age 3 or 4, they will not yet be competent in any language, and Spanish-language instruction may hinder competence in the native language. In the study area, the preschool teachers from the 1950's onwards in Juchitán, and 20-30-years later in the outlying communities, discouraged speaking Zapotec, both because of its perceived cultural inferiority and because they were unable to speak it and thus communicate with the children [44]. The mothers, in order to avoid conflict with the teachers, started speaking Spanish at home. However, political and cultural movements have played a significant role in counteracting these influences, leading to a relatively high average competence in Zapotec, compared to other indigenous peoples and communities [44,81,82].

### Interactions between variables

The most important factor, occupation, is modulated by other factors, such as locality and schooling. People from small towns and villages, even if their work does not include direct contact with natural resources, will encounter plants in their daily lives anyway (for example, on the hills in the center of their communities, with names that indicate their importance, Cerro del Tigre and Cerro Santa Cruz). They are immersed in a cultural environment that includes frequent references to plants. Also, many people employed in the non-farm activities still farm part-time, either for economic reasons or out of respect for tradition and heritage. In Juchitán, modernity is more established as a desirable worldview.

The higher level of farmers’ knowledge in San Blas Atempa, the intermediate site, can be explained by slightly tighter social cohesion: the persistence of regular, decision-making assemblies and of the communal authorities is an expression of this. The attitude towards the land is also different from the other two sites, perhaps because land issues are regularly discussed in the assemblies. A “Consejo de Vigilancia” (a Guard Committee) makes regular inspection rounds of the territory; several of our interviewees had taken part in this activity at one time or the other. However, even though the knowledge level is slightly higher in San Blas, knowledge loss is also faster. This slightly accelerated loss is perhaps due to the oil refinery, which introduced another way of life.

The data underscore the relatively strong relationship between locality and plant knowledge, even for people in non-agricultural jobs. These local political, economic and cultural influences reflect intracultural differences due to history and geography. Once communities differentiate and evolve, they may respond differently to outside pressures. This leads to the intracultural variation observed in this work.

## Conclusions

All of the studied factors—economic activity, age, Zapotec language competence, formal schooling, and locality—influenced botanical (and, by inference, ecological) knowledge. Together, they explained 78% of the variation in the plant knowledge of Zapotec men. However, economic activity was by far the most important determinant of this type of knowledge. This, together with the relative significance of the other factors, shows that exposure is the main factor influencing knowledge of the domain, in this case nature. The significant interaction of economic activity with locality reflects intracultural differences due to variation in local processes.

## Supporting Information

S1 Fig An example of the form used for the interviews. (JPG)

S2 Fig Letters by legal representatives of the communities indicating that no permit was required for the investigation. (PDF)

S1 Table Results of the t-test for independent samples, showing the statistical significance of differences between groups of economic activities, as defined in Table 3. (DOC)

S2 Table Comparison of plant knowledge between linguistic competence groups with an ANOVA (p = .000). The differences between groups were statistically highly significant.
(DOC)

S3 Table The relationship between various knowledge levels and the sociodemographic variables. Analyzed with a general linear analysis of between-subject effects and its statistical significance. It shows the relative contribution of each variable (Partial Eta Squared).
(DOC)

S4 Table The anonymized, raw data of the interviews in Excel. (XLS)
