# Supplementary figures and images for: Intracultural Differences in Local Botanical Knowledge and Knowledge Loss among the Mexican Isthmus Zapotecs
Source: PLoS One. 2016 Mar 17;11(3):e0151693. doi: 10.1371/journal.pone.0151693 (PMC4795621; doi:10.1371/journal.pone.0151693)

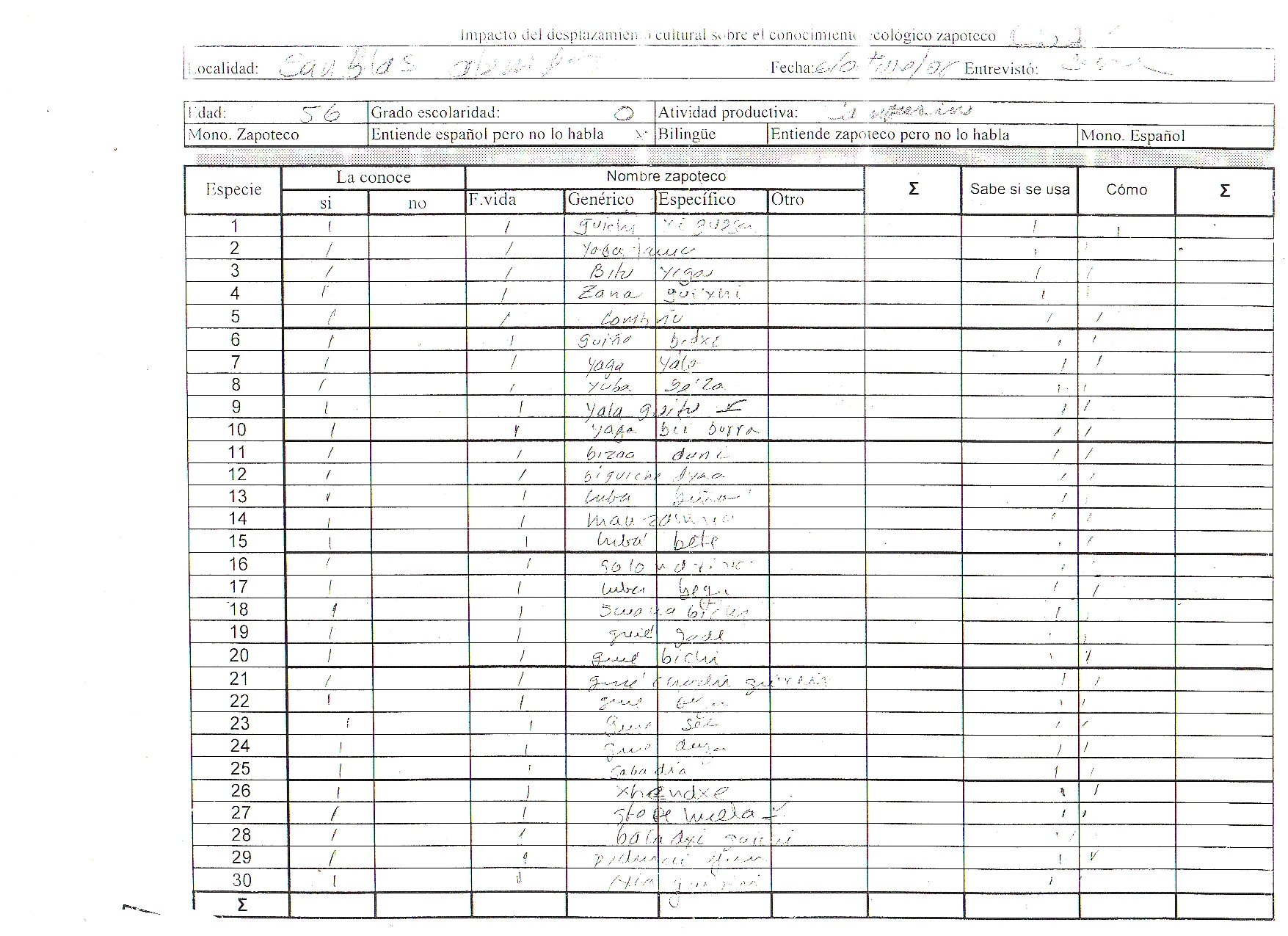

Supplement: S1 Fig — (JPG) [file pone.0151693.s001.jpg]
