# Supplementary material for: Intracultural Differences in Local Botanical Knowledge and Knowledge Loss among the Mexican Isthmus Zapotecs
Source: PLoS One. 2016 Mar 17;11(3):e0151693. doi: 10.1371/journal.pone.0151693 (PMC4795621; doi:10.1371/journal.pone.0151693)
Supplement: S2 Fig — (PDF) [file pone.0151693.s002.pdf]

SANTA MARÍA
XADANI
H. AYUNTAMIENTO 2014 | 2016

**DEPENDENCIA:** H. AYUNTAMIENTO MUNICIPAL
**SECCIÓN:** SINDICATURA MUNICIPAL
**N° OFICIO:** SM/134/2015
**EXPEDIENTE:** 2014-2016
**ASUNTO:** CONSTANCIA.

A QUIEN CORRESPONDA:

El que suscribe C. **LIC. RUBISEL SANTIAGO GUERRA**, con el carácter de Síndico Municipal y representante legal, que me confiere el Artículo 71 de la Ley Orgánica Municipal del Estado de Oaxaca del H. Ayuntamiento de este municipio:

H A G O C O N S T A R:

Que el C. ALFREDO SAYNES VASQUEZ, cuyos documentos presentados en esta Sindicatura Municipal, coinciden con los de la persona, Quién tiene su domicilio conocido s/n, Juchitán de Zaragoza Oaxaca, C.P. 70000, compareció ante esta Sindicatura Municipal, quién manifestó haber realizado un trabajo de INVESTIGACIÓN ETNOBIOLOGICA, durante los años 2005 a 2010 en esta Municipalidad, mismas que acredita con documento consistente en 10 fojas útiles de la investigación realizada, así mismo manifiesto en calidad de Síndico Municipal que para la realización de trabajos de investigación en nuestro Municipio que es una Comunidad Indígena Zapoteca no se requiere previa autorización o permiso alguno de esta Autoridad o alguna otra, por no estar regulada en un texto normativo .- Doy Fe.- - - - - - - - - - - - - - - - - - - - - - - - - - - - - - - - - - - - - - - - - - - - - - - - - - - - - - - - - - - - - - - - ---

A petición del interesado y para los usos legales a que haya lugar, se expide la presente Constancia, en el municipio de Santa María Xadani, Oaxaca, a los Veinticuatro días del mes de Septiembre del Año Dos Mil Quince. - - - - - - - - - - - - - - - - - - - - - - - - - - - - - - - ----

A T E N T A M E N T E
"SUFRAGIO EFECTIVO, NO REELECCION"
"EL RESPETO AL DERECHO AJENO, ES LA PAZ"
EL SÍNDICO MUNICIPAL

*C.LIC. RUBISEL SANTIAGO GUERRA*

ESTADOS UNIDOS MEXICANOS
SINDICATURA MUNICIPAL
Mpio. Santa María Xadani
Dtto. Juchitán, Oax
2014 - 2016

# H. AYUNTAMIENTO MUNICIPAL CONSTITUCIONAL DE LA
## Heroica Ciudad de Juchitán de Zaragoza, Oax.
## 2014-2016

H. Ayuntamiento Constitucional de la Heroica Ciudad de Juchitán de Zaragoza, Oax. 2014-2016

DEPENDENCIA: **SECRETARIA MUNICIPAL**
No. DE OFICIO: **MJZ/SM/263/2015**
ASUNTO: **CONSTANCIA**

**A QUIEN CORRESPONDA:**

**EMILIO MONTERO PEREZ,** Secretario Municipal del H. Ayuntamiento de la Heroica Ciudad de Juchitán de Zaragoza, Oaxaca, en ejercicio de las facultades que me confiere el Artículo 92, Fracción VI, de la Ley Orgánica Municipal del Estado de Oaxaca y el artículo 114 fracción VII del Bando de policía y gobierno de la H. Ciudad de Juchitán de Zaragoza, Oaxaca en vigor, a usted con el debido respeto hago:

**C O N S T A R:**

Que el **C. ALFREDO SAYNES VASQUEZ,** cuyo documentos presentados en esta Secretaria Municipal, coinciden con los de la persona, Quien tiene su domicilio conocido S/n, Juchitán de Zaragoza, Oaxaca, C.P. 70000, Compareció en esta oficina Municipal, quien manifestó haber realizado un trabajo de **INVESTIGACION ETNOBIOLOGICA** durante los años 2005 a 2010 en esta municipalidad, misma que acredita con documento consistente en 10 fojas útiles de la investigación realizada, así mismo manifiesto en mi calidad de Secretario Municipal que para la realización de trabajos de investigación en nuestro Municipio que es una comunidad Indígena Zapoteca no se requiere previa autorización o permiso alguno de esta autoridad o alguna otra, por no estar regulada en un texto Normativo.- Doy Fe, por la que a petición de la parte interesada, se extiende la presente Constancia en la Heroica Ciudad de Juchitán de Zaragoza Oaxaca. Para el efecto legal correspondiente, a los veinticuatro días del mes de septiembre del año dos mil quince.------------------------------ -

ATENTAMENTE
SUFRAGIO EFECTIVO, NO REELECCION
"EL RESPETO AL DERECHO AJENO ES LA PAZ"

SECRETARIA MUNICIPAL
Mpio. Heroica Ciudad de Juchitán de Zaragoza
Dtto. de Juchitán, Oax.
2014-2016

------------------------------------------------
EMILIO MONTERO PÉREZ
SECRETARIO MUNICIPAL

CC.ARCHIVO/EXPEDIENTE
EMP/LPV

H. AYUNTAMIENTO MUNICIPAL
CONSTITUCIONAL
VILLA DE SAN BLAS ATEMPA, OAXACA
TRIENIO 2014 - 2016

| | |
|---|---|
| DEPENDENCIA | H. AYUNTAMIENTO MUNICIPAL CONSTITUCIONAL |
| ÁREA | SECRETARIO MUNICIPAL |
| OFICIO N° | SM/115/2015 |
| EXPEDIENTE | 2015 |

**ASUNTO: CONSTANCIA DE INVESTIGACION**

A QUIEN CORRESPONDA:

El que suscribe **C. VICTOR CARRASCO QUIROZ,** Secretario Municipal Constitucional del H. Ayuntamiento Municipal Constitucional de la Villa de San Blas Atempa, Distrito Judicial de Santo Domingo, Tehuantepec, Estado de Oaxaca, con fundamento en Titulo Quinto, Capítulo I, Articulo 92 en la Ley Orgánica Municipal para el Estado de Oaxaca en vigor.

# HAGO CONSTAR

Que el (a) C. ALFREDO SAYNES VASQUEZ, es originario y vecino de Juchitán de Zaragoza, Oaxaca, C.P. 70 000, con domicilio conocido s/n, hago constar que realizo un trabajo **de** INVESTIGACION ETNOBIOLOGICA, durante los años 2005 a 2010 en la cabecera de este Municipio y sus 5 Agencias, misma que acredita con documento consistente en 10 fojas útiles de la investigación realizada, así mismo manifiesto en mi calidad de Secretario Municipal que para realización de trabajos de investigación en nuestro Municipio que es una comunidad Indígena Zapoteca no se requiere previa autorización o permiso alguno de esta autoridad o alguna otra, por no estar regulada en un texto Normativo. y es de nacionalidad mexicana.

A petición de la parte interesada y para los efectos legales a que haya lugar, se extiende la presente en esta Villa de San Blas Atempa, Oaxaca, a veinticinco días del mes de septiembre del dos mil quince.

A T E N T A M E N T E
"SUFRAGIO EFECTIVO, NO REELECCION "
"EL RESPETO AL DERECHO AJENO ES LA PAZ"
SECRETARIO MUNICIPAL

SECRETARIA MUNICIPAL
Municipio Villa de San Blas Atempa
Distrito. Tehuantepec
Oaxaca 2014 2016

C. VICTOR CARRASCO QUIROZ

c.c.p. Expediente.
c.c.p. minutario
