# Supplementary material for: Intracultural Differences in Local Botanical Knowledge and Knowledge Loss among the Mexican Isthmus Zapotecs
Source: PLoS One. 2016 Mar 17;11(3):e0151693. doi: 10.1371/journal.pone.0151693 (PMC4795621; doi:10.1371/journal.pone.0151693)
Supplement: S1 Table — (DOC) [file pone.0151693.s003.doc]

**S1 Table.** Results of the *t*-test for independent samples, showing the statistical significance of differences between groups of economic activities, as defined in Table 3.

| Competence | | Levene's Test for Equality of Variances | | t-test for Equality of Means | | |
| --- | --- | --- | --- | --- | --- | --- |
|  |  | F | Sig. | t | df | Sig. (2-tailed) |
| Visual recognition | Equal variances assumed | .758 | .385 | 21.367 | 298 | .000 |
|  | Equal variances not assumed |  |  | 21.367 | 290.726 | .000 |
| Plant form | Equal variances assumed | 1.163 | .282 | 21.383 | 298 | .000 |
|  | Equal variances not assumed |  |  | 21.383 | 288.641 | .000 |
| Generic name | Equal variances assumed | 12.810 | .000 | 20.764 | 298 | .000 |
|  | Equal variances not assumed |  |  | 20.764 | 274.182 | .000 |
| Specific name | Equal variances assumed | 12.551 | .000 | 20.793 | 298 | .000 |
|  | Equal variances not assumed |  |  | 20.793 | 273.226 | .000 |
| Use | Equal variances assumed | 28.678 | .000 | 21.402 | 298 | .000 |
|  | Equal variances not assumed |  |  | 21.402 | 253.545 | .000 |
| Global Index | Equal variances assumed | .933 | .335 | 23.790 | 298 | .000 |
|  | Equal variances not assumed |  |  | 23.790 | 295.203 | .000 |
