# Supplementary material for: Intracultural Differences in Local Botanical Knowledge and Knowledge Loss among the Mexican Isthmus Zapotecs
Source: PLoS One. 2016 Mar 17;11(3):e0151693. doi: 10.1371/journal.pone.0151693 (PMC4795621; doi:10.1371/journal.pone.0151693)
Supplement: S2 Table — The differences between groups were statistically highly significant. (DOC) [file pone.0151693.s004.doc]

**S2 Table.** Comparison of plant knowledge between linguistic competence groups with an ANOVA (p = .000). The differences between groups were statistically highly significant.

| Competence |  | Sum of Squares | df | Mean Square | F | Sig. |
| --- | --- | --- | --- | --- | --- | --- |
| Visual | Between Groups | 4379.295 | 3 | 1459.765 | 26.191 | .000 |
|  | Within Groups | 16497.871 | 296 | 55.736 |  |  |
|  | Total | 20877.166 | 299 |  |  |  |
| Plant form | Between Groups | 4990.403 | 3 | 1663.468 | 28.564 | .000 |
|  | Within Groups | 17238.083 | 296 | 58.237 |  |  |
|  | Total | 22228.486 | 299 |  |  |  |
| Generic name | Between Groups | 6538.713 | 3 | 2179.571 | 36.089 | .000 |
|  | Within Groups | 17876.554 | 296 | 60.394 |  |  |
|  | Total | 24415.267 | 299 |  |  |  |
| Specific name | Between Groups | 8071.770 | 3 | 2690.590 | 37.396 | .000 |
|  | Within Groups | 21296.785 | 296 | 71.949 |  |  |
|  | Total | 29368.556 | 299 |  |  |  |
| Use | Between Groups | 3801.185 | 3 | 1267.062 | 30.691 | .000 |
|  | Within Groups | 12220.028 | 296 | 41.284 |  |  |
|  | Total | 16021.213 | 299 |  |  |  |
| Global Index | Between Groups | 1879.785 | 3 | 626.595 | 33.072 | .000 |
|  | Within Groups | 5608.189 | 296 | 18.947 |  |  |
|  | Total | 7487.974 | 299 |  |  |  |
