# Supplementary material for: Intracultural Differences in Local Botanical Knowledge and Knowledge Loss among the Mexican Isthmus Zapotecs
Source: PLoS One. 2016 Mar 17;11(3):e0151693. doi: 10.1371/journal.pone.0151693 (PMC4795621; doi:10.1371/journal.pone.0151693)
Supplement: S3 Table — Analyzed with a general linear analysis of between-subject effects and its statistical significance. It shows the relative contribution of each variable (Partial Eta Squared). (DOC) [file pone.0151693.s005.doc]

**S3 Table.** Relationship between various knowledge levels and the sociodemographic variables, analyzed with a general linear analysis of between-subject effects and its statistical significance. It shows the relative contribution of each variable (Partial Eta Squared)

**Level 1: Visual recognition**

Dependent Variable: Visual Recognition

| Source | Type III Sum of Squares | df | Mean Square | F | Sig. | Partial Eta Squared |
| --- | --- | --- | --- | --- | --- | --- |
| Corrected Model | 14674.843a | 8 | 1834.355 | 86.064 | .000 | .703 |
| Intercept | 2001.059 | 1 | 2001.059 | 93.885 | .000 | .244 |
| Age | 129.885 | 1 | 129.885 | 6.094 | .014 | .021 |
| Schooling | 285.352 | 1 | 285.352 | 13.388 | .000 | .044 |
| Speak Zapotec | 84.590 | 1 | 84.590 | 3.969 | .047 | .013 |
| Locality | 523.381 | 2 | 261.690 | 12.278 | .000 | .078 |
| Economic Activity | 4954.597 | 1 | 4954.597 | 232.459 | .000 | .444 |
| Locality * Economic Activity | 810.829 | 2 | 405.415 | 19.021 | .000 | .116 |
| Error | 6202.323 | 291 | 21.314 |  |  |  |
| Total | 293683.591 | 300 |  |  |  |  |
| Corrected Total | 20877.166 | 299 |  |  |  |  |

a. R Squared = .703 (Adjusted R^2^ = .695)

**Level 2: Plant life form**

Dependent Variable: Plant life form

| Source | Type III Sum of Squares | df | Mean Square | F | Sig. | Partial Eta Squared |
| --- | --- | --- | --- | --- | --- | --- |
| Corrected Model | 16016.871a | 8 | 2002.109 | 93.794 | .000 | .721 |
| Intercept | 1820.976 | 1 | 1820.976 | 85.309 | .000 | .227 |
| Age | 157.014 | 1 | 157.014 | 7.356 | .007 | .025 |
| Schooling | 407.928 | 1 | 407.928 | 19.110 | .000 | .062 |
| Speak Zapotec | 116.253 | 1 | 116.253 | 5.446 | .020 | .018 |
| Locality | 642.905 | 2 | 321.452 | 15.059 | .000 | .094 |
| Economic Activity | 4975.710 | 1 | 4975.710 | 233.101 | .000 | .445 |
| Locality * Economic Activity | 942.961 | 2 | 471.481 | 22.088 | .000 | .132 |
| Error | 6211.615 | 291 | 21.346 |  |  |  |
| Total | 287799.689 | 300 |  |  |  |  |
| Corrected Total | 22228.486 | 299 |  |  |  |  |

a. R Squared = .721 (Adjusted R^2^ = .713)

**Level 3: Generic name**

Dependent Variable: Generic name

| Source | Type III Sum of Squares | df | Mean Square | F | Sig. | Partial Eta Squared |
| --- | --- | --- | --- | --- | --- | --- |
| Corrected Model | 18539.941a | 8 | 2317.493 | 114.783 | .000 | .759 |
| Intercept | 649.290 | 1 | 649.290 | 32.159 | .000 | .100 |
| Age | 409.442 | 1 | 409.442 | 20.279 | .000 | .065 |
| Schooling | 665.945 | 1 | 665.945 | 32.984 | .000 | .102 |
| Speak Zapotec | 393.805 | 1 | 393.805 | 19.505 | .000 | .063 |
| Locality | 586.789 | 2 | 293.394 | 14.532 | .000 | .091 |
| Economic Activity | 4531.428 | 1 | 4531.428 | 224.438 | .000 | .435 |
| Locality * Economic Activity | 1429.832 | 2 | 714.916 | 35.409 | .000 | .196 |
| Error | 5875.326 | 291 | 20.190 |  |  |  |
| Total | 226192.249 | 300 |  |  |  |  |
| Corrected Total | 24415.267 | 299 |  |  |  |  |

a. R Squared = .759 (Adjusted R^2^ = .753)

**Level 4: Specific name**

Dependent Variable: Specific name

| Source | Type III Sum of Squares | df | Mean Square | F | Sig. | Partial Eta Squared |
| --- | --- | --- | --- | --- | --- | --- |
| Corrected Model | 22385.282a | 8 | 2798.160 | 116.602 | .000 | .762 |
| Intercept | 430.162 | 1 | 430.162 | 17.925 | .000 | .058 |
| Age | 515.309 | 1 | 515.309 | 21.473 | .000 | .069 |
| Schooling | 987.116 | 1 | 987.116 | 41.134 | .000 | .124 |
| Speak Zapotec | 397.844 | 1 | 397.844 | 16.579 | .000 | .054 |
| Locality | 931.261 | 2 | 465.630 | 19.403 | .000 | .118 |
| Economic Activity | 5172.981 | 1 | 5172.981 | 215.563 | .000 | .426 |
| Locality * Economic Activity | 1395.501 | 2 | 697.751 | 29.076 | .000 | .167 |
| Error | 6983.273 | 291 | 23.998 |  |  |  |
| Total | 194839.662 | 300 |  |  |  |  |
| Corrected Total | 29368.556 | 299 |  |  |  |  |

a. R Squared = .762 (Adjusted R^2^ = .756)

**Level 5: Use**

Dependent Variable: Use

| Source | Type III Sum of Squares | df | Mean Square | F | Sig. | Partial Eta Squared |
| --- | --- | --- | --- | --- | --- | --- |
| Corrected Model | 12150.893a | 8 | 1518.862 | 114.200 | .000 | .758 |
| Intercept | 949.464 | 1 | 949.464 | 71.388 | .000 | .197 |
| Age | 249.167 | 1 | 249.167 | 18.734 | .000 | .060 |
| Schooling | 574.464 | 1 | 574.464 | 43.193 | .000 | .129 |
| Speak Zapotec | 78.220 | 1 | 78.220 | 5.881 | .016 | .020 |
| Locality | 663.129 | 2 | 331.564 | 24.930 | .000 | .146 |
| Economic Activity | 2950.224 | 1 | 2950.224 | 221.820 | .000 | .433 |
| Locality * Economic Activity | 567.696 | 2 | 283.848 | 21.342 | .000 | .128 |
| Error | 3870.321 | 291 | 13.300 |  |  |  |
| Total | 171848.806 | 300 |  |  |  |  |
| Corrected Total | 16021.213 | 299 |  |  |  |  |

a. R Squared = .758 (Adjusted R^2^ = .752)
